# Application of machine learning algorithms to predict lymph node metastasis in gastric neuroendocrine neoplasms

**DOI:** 10.1016/j.heliyon.2023.e20928

**Published:** 2023-10-18

**Authors:** Lu Liu, Wen Liu, Zhenyu Jia, Yao Li, Hongyu Wu, Shuting Qu, Jinzhou Zhu, Xiaolin Liu, Chunfang Xu

**Affiliations:** a Department of Gastroenterology, The First Affiliated Hospital of Soochow University, Suzhou, China

**Keywords:** Neuroendocrine neoplasms, Machine learning, Predictive model, Lymph node metastasis

## Abstract

**Background:**

Neuroendocrine neoplasms (NENs) are tumors that originate from secretory cells of the diffuse endocrine system and typically produce bioactive amines or peptide hormones. This paper describes the development and validation of a predictive model of the risk of lymph node metastasis among gastric NEN patients based on machine learning platform.

**Methods:**

In this investigation, data from 1256 patients were used, of whom 119 patients from the First Affiliated Hospital of Soochow University in China and 1137 cases from the surveillance epidemiology and end results (SEER) database were combined. Six machine learning algorithms, including the logistic regression model (10.13039/501100009319LR), random forest (RF), decision tree (DT), Naive Bayes (10.13039/100004395NB), support vector machine (SVM), and k-nearest neighbor algorithm (KNN), were used to build the predictive model. The performance of the models was evaluated using the area under the receiver operating characteristic curve (AUC), accuracy, sensitivity, and specificity.

**Results:**

Among the 1256 patients with gastric NENs, 276 patients (21.97 %) developed lymph node metastasis. T stage, tumor size, degree of differentiation, and sex were predictive factors of lymph node metastasis. The RF model achieved the best predictive performance among the six machine learning models, with an AUC, accuracy, sensitivity, and specificity of 0.81, 0.78, 0.76, and 0.82, respectively.

**Conclusion:**

The RF model provided the best prediction and can help physicians determine the lymph node metastasis risk of gastric NEN patients to formulate individualized medical strategies.

## 1 Introduction

Neuroendocrine neoplasms (NENs) are heterogeneous malignancies that are thought to originate from neuroendocrine precursor cells or arise due to neuroendocrine transdifferentiation of organ-specific epithelial cell types, with an annual age-adjusted incidence of approximately 3.6 cases per 100,000 individuals [1,2]. Approximately two-thirds of NENs occur in the gastroenteropancreatic system, and gastric NENs account for approximately 6.9 %–23 % of all gastroenteropancreatic neuroendocrine neoplasms [[3], [4], [5], [6], [7]]. In recent years, with the popularization of physical examination and the improvement of detection methods (CT and endoscopy), the detection rate of gastric NENs has increased from 7-fold to 10-fold [8,9]. Gastric NENs are derived from enterochromaffin cells and can be subdivided into three types. Gastric NEN1 and gastric NEN2 are both associated with hypergastrinaemia, the former in the context of chronic autoimmune gastritis, and the latter in the context of gastrinomas [10,11]. Gastric NEN3 has normal gastrin concentrations but displays more aggressive clinical behavior. Gastric NEN1 and gastric NEN2 are more likely to present as multiple small polyp lesions, whereas gastric NEN3 are usually solitary, and the lesion is large (most >2 cm in diameter), presenting as a polypoid mass or ulcer [12].

There are different opinions concerning the endoscopic dissection of gastric NENs, with some scholars arguing that gastric NEN1 less than 2 cm in size, no more than six in number, and confined to the mucosa and submucosa can undergo endoscopic resection [13]. However, some researchers found that a tumor size ≥1 cm is linked to a higher risk of lymph node metastasis (LNM) in gastric NEN1 patients [14]. Additionally, gastric NEN3, with lesions less than 2 cm in size and no evidence of lymphovascular invasion, for which radical resection was often required in the past, can now be treated with endoscopic mucosal resection (EMR) or endoscopic submucosal dissection (ESD), showing no recurrence [15]. An accurate prediction of the likelihood of LNM in gastric NEMs can therefore better inform therapeutic decision-making as LNM is a key factor in determining the administration of endoscopic therapy. Thus, we decided to construct and validate a model based on clinicopathological information to identify those at high risk of LNM of gastric NENs.

Most recently, artificial intelligence has been applied in oncology to construct models, such as in the prediction of breast cancer and lung cancer survival [16,17]. Machine learning can automatically learn from a large amount of data, establishing the implicit relationship between variables and outcomes, and then a highly effective model can be constructed to predict the outcome of other data. Six machine learning algorithms were used in this study to predict the LNM among gastric NEN patients.

## 2 Methods

### 2.1 Study population and data classification

**Fig. 1:**
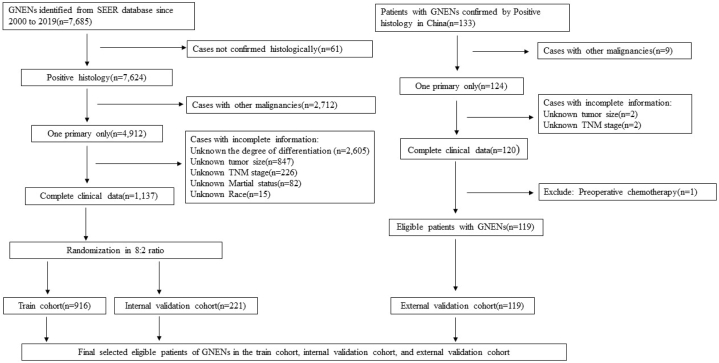
The data collection flow diagram. The data from 1137 gastric NEN patients of SEER were randomly assigned to the train and internal validation cohorts in an 8:2 ratio, and 119 patients from the First Affiliated Hospital of Soochow University were used as an external validation cohort. GNEN, gastric neuroendocrine neoplasms.

Patient data were obtained between 2000 and 2019 from the SEER database, which contains patient information and cancer characteristics (http://www.seer.cancer.gov) [18]. Cases were selected based on the primary site code (C16.0-C16.9, stomach) and Third Edition (ICD-*O*-3) histology codes (8013, Large cell neuroendocrine carcinoma; 8153, Gastrinoma; 8240, Carcinoid tumor; 8241, Enterochromaffin cell carcinoid; 8242, Enterochromaffin-like cell tumor; 8244, Mixed adenoneuroendocrine carcinoma; 8245, adenocarcinoid tumor; 8246, neuroendocrine carcinoma; and 8249, atypical carcinoid tumor; 8156, Somatostatinoma). Exclusion criteria were: (1) cases with a history of other malignancies; (2) cases without complete clinical data. Overall, 1137 gastric NEN patients without missing values were included, as well as another 119 gastric NEN patients enrolled via medical records retrospectively from the First Affiliated Hospital of Soochow University between 2010 and 2020. Patients were included if the diagnosis of gastric NENs was pathologically confirmed and they did not have other primary tumors. Data information retrieved included patient characteristics (race, sex, and age at diagnosis), tumor demographics (primary site, degree of differentiation, T stage, N stage, lymph node, and organ metastases), and treatment data (surgery, chemotherapy, and radiation therapy). All these patients were diagnosed by histopathology from 2011 to 2020. The case screening process is described in Fig. 1.

### 2.2 Development and validation of prediction models

The cohort from the SEER database was included in the training set and the internal validation set and the cohort from the First Affiliated Hospital of Soochow University was included in the external validation set. Missing values were detected using the mice package and ﬁlled with predictive mean matching. Six machine-learning algorithms were employed to predict the lymph noder metastasis (10.13039/501100015347LNM) in patients with gastric NENs including the logistic regression model (10.13039/501100009319LR), random forest (RF), decision tree (DT), Naive Bayes (10.13039/100004395NB), support vector machine (SVM), and k-nearest neighbor algorithm (KNN). The AUCs were calculated to identify the predictive ability of the six machine learning models in the test cohort and a correlation heat map of the variables was produced to evaluate the correlation of each predictor.

### 2.3 Model Interpretation

Shapley Additive Explanations (SHAP) were implemented as an additive feature attribution method to visualize the association between features and the metastasis status. The horizontal axis represents the Shapley value, for which a value greater than 0 indicates a positive contribution to the LNM of gastric NENs. The left longitudinal coordinate expresses the important order of features in reverse. The right longitudinal coordinate indicates the features’ value from low to high. The performance of the machine learning models was evaluated by the ROC, model accuracy, sensitivity, and specificity. A P-value <0.05 was considered statistically significant.

### 2.4 Statistical analysis

All statistical analyses were performed in R software (version 4.1.0) (http://www.R-project.org) and Python (version 3.8, Python Software Foundation). The training and internal validation groups were extracted from the SEER database using SEER statistical software (version 8.3.6) and randomly assigned to the developing and internal validation cohorts at a ratio of 8:2. Training and validation sets were used to build the models, and the external validation cohort was used for external validation and evaluation.

## 3 Results

### 3.1 Demographic baseline characteristics

The 1137 patients from the SEER database were divided into training and internal validation sets at a ratio of 8:2, and 119 patients from the First Affiliated Hospital of Soochow University were used as the external validation set. The differences between the SEER database and the hospital patients according to the presence of lymph node metastases are shown in Table 1.

**Table 1:** Demographic and clinical characteristics of patients in the SEER database and external validation group. SEER, surveillance epidemiology and end results; T, t stage; M, metastasis; LNM：lymph node metastasis.

Variable		SEER data (n = 1137)			External validation dataset (n = 119)		
	Group	LNM No [n (%)]	LNM Yes [n (%)]	P	LNM N0 [n (%)]	LNM Yes [n (%)]	P
Sex（%）	Female	601 (64.5)	81 (39.5)	<0.001	21 (43.8)	14 (19.7)	0.009
	Male	331 (35.5)	124 (60.5)		27 (56.2)	57 (80.3)	
Age (mean (SD))		59.33 (13.20)	62.48 (13.87)	0.002	59.23 (12.53)	66.08 (8.99)	0.001
Race(%)	white	741 (79.5)	153 (74.6)	0.286			
	black	120 (12.9)	34 (16.6)				
	others	71 (7.6)	18 (8.8)				
Marital status (%)	unmarried	384 (41.2)	73 (35.6)	0.162			
	married	548 (58.8)	132 (64.4)				
Primary site (%)	Cardia/Fundus	148 (15.9)	59 (28.8)	<0.001	15 (31.2)	43 (60.6)	0.005
	Body	315 (33.8)	32 (15.6)		20 (41.7)	12 (16.9)	
	Antrum/Pylorus	100 (10.7)	28 (13.7)		7 (14.6)	12 (16.9)	
	Overlapping	26 (2.8)	14 (6.8)		4 (8.3)	4 (5.6)	
	Greater/Lesser curvature/Stomach, NOS	343 (36.8)	72 (35.1)		2 (4.2)	0 (0.0)	
Differentiation (%)	Well	695 (74.6)	57 (27.8)	<0.001	24 (50.0)	0 (0.0)	<0.001
	moderately	163 (17.5)	33 (16.1)		0 (0.0)	3 (4.2)	
	Poorly + undifferentiated	74 (7.9)	115 (56.1)		24 (50.0)	68 (95.8)	
Size (%)	≤1	597 (64.1)	10 (4.9)	<0.001	18 (37.5)	0 (0.0)	<0.001
	＞1 and ≤ 2	178 (19.1)	28 (13.7)		3 (6.2)	2 (2.8)	
	＞2 and ≤ 5	109 (11.7)	90 (43.9)		19 (39.6)	42 (59.2)	
	＞5	48 (5.2)	77 (37.6)		8 (16.7)	27 (38.0)	
T (%)	Tis	37 (4.0)	1 (0.5)	<0.001	0	0	<0.001
	T1	542 (58.2)	6 (2.9)		18 (37.5)	0 (0.0)	
	T2	267 (28.6)	64 (31.2)		8 (16.7)	4 (5.6)	
	T3	46 (4.9)	72 (35.1)		17 (35.4)	52 (73.2)	
	T4	40 (4.3)	62 (30.2)		5 (10.4)	15 (21.1)	
M(%)	M0	879 (94.3)	132 (64.4)	<0.001	45 (93.8)	61 (85.9)	0.296
	M1	53 (5.7)	73 (35.6)		3 (6.2)	10 (14.1)	

### 3.2 Univariate and multivariate logistic regression analysis

**Fig. 2:**
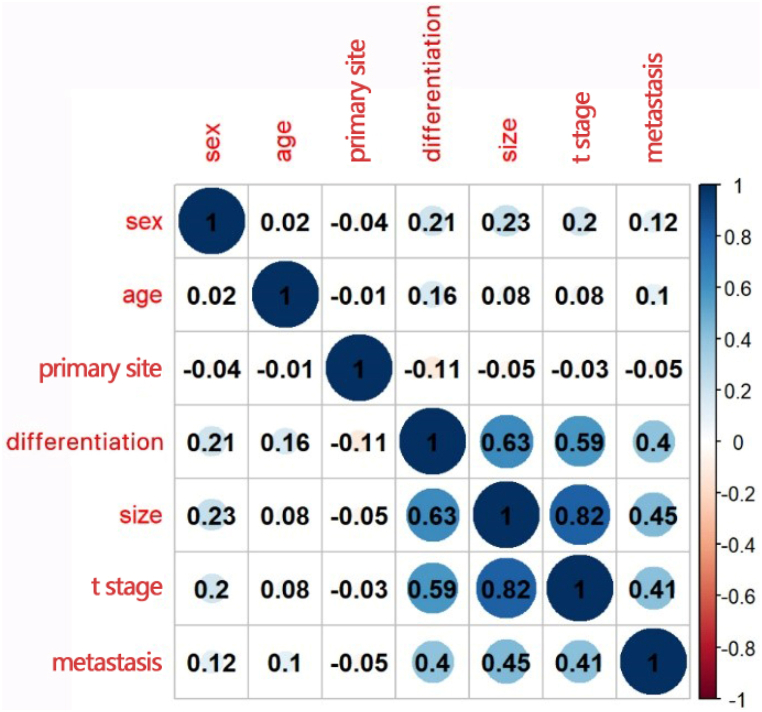
Results of the correlation analysis of all variables. Blue indicates a positive correlation and red indicates a negative correlation. (For interpretation of the references to colour in this figure legend, the reader is referred to the Web version of this article.)

From the logistic regression analysis, sex, primary site, tumor size, and T and M stage were significantly associated with LNM in gastric NENs (P < 0.05). Significant variables were included the multivariate logistic regression analysis, revealing that sex (OR: 1.968, 95 % CI: 1.241−3.139), degree of differentiation (OR: 2.546, 95 % CI: 1.304−4.979), T (OR: 11.925, 95 % CI: 1.872−235.985) and M stage (OR: 2.636, 95 % CI: 1.487−4.716) were independent risk factors for LNM in gastric NENs (Table 2). The correlation heatmap (Fig. 2) revealed a weak relationship between age and other variables.

**Table 2:** The logistic regression of the variables. T, t stage; M, metastasis.

Variables	Univariate analysis		Multivariate analysis	
	OR (95 % CI)	p value	OR (95 % CI)	p value
Sex				
Female	Ref		Ref	Ref
Male	2.960 [2.106, 4.185]	<0.001	1.968 [1.241,3.139]	0.004
Age (mean (SD))	1.017 [1.004, 1.031]	0.009	1.004 [0.987,1.020]	0.661
Race				
White	Ref		Ref	Ref
Black	1.731 [1.081, 2.715]	0.019	1.425 [0.749,2.680]	0.274
Others	1.299 [0.689, 2.314]	0.395	0.686 [ 0.306,1.491]	0.350
Marital status (%)				
Unmarried	Ref		Ref	Ref
Married	1.202 [0.853, 1.706]	0.297	/	/
Primary site				
Cardia/Fundus	Ref		Ref	Ref
Body	0.256 [0.148, 0.434]	<0.001	1.167 [0.550,2.484]	0.687
Antrum/Pylorus	0.970 [0.550, 1.689]	0.915	1.204 [0.555,2.625]	0.638
Overlapping	1.442 [0.623, 3.205]	0.377	1.992 [ 0.643,6.265]	0.233
Greater/Lesser curvature/Stomach, NOS	0.583 [0.376, 0.908]	0.016	1.809 [0.968,3.453]	0.067
Differentiation				
Well	Ref		Ref	Ref
Moderately	2.867 [1.724, 4.716]	<0.001	1.672 [0.901,3.066]	0.099
Poorly + Undifferentiated	20.524 [13.237, 32.371]	<0.001	2.546 [1.304,4.979]	0.006
Size				
≤1	Ref		Ref	Ref
＞1 and ≤ 2	8.221 [3.948, 18.394]	<0.001	1.186 [0.366,4.636]	0.789
＞2 and ≤ 5	40.160 [20.884, 85.499]	<0.001	2.813 [0.898,10.741]	0.097
＞5	90.745 [44.413, 203.514]	<0.001	2.540 [0.711,10.670]	0.171
T				
Tis	Ref		Ref	Ref
T1	0.425 [0.069, 8.152]	0.435	0.437 [0.067,8.717]	0.463
T2	7.458 [1.543, 134.277]	0.051	2.760 [0.458,53.474]	0.357
T3	51.971 [10.411, 945.916]	<0.001	11.925 [1.872,235.985]	0.027
T4	55.000 [10.965, 1002.708]	<0.001	8.340 [1.289,165.955]	0.060
M				
M0	Ref		Ref	Ref
M1	10.945 [7.033, 17.244]	<0.001	2.636 [ 1.487,4.716]	<0.001

### 3.3 Models performance

**Fig. 3:**
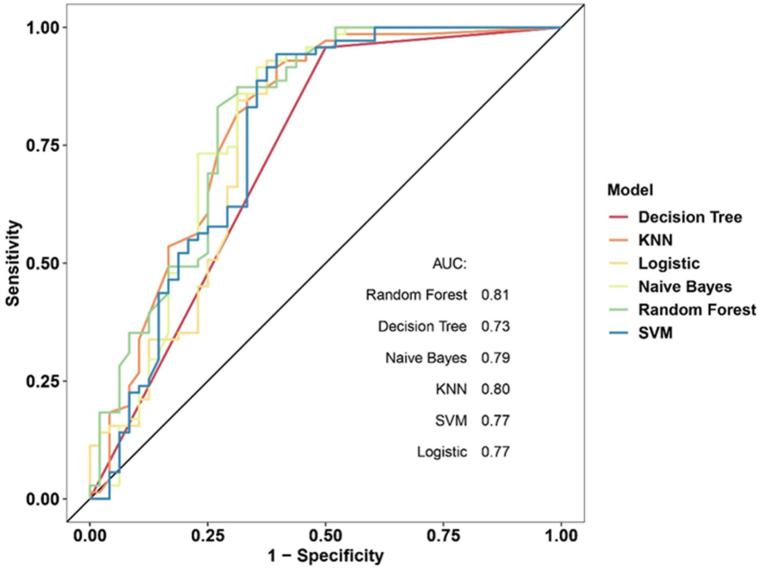
ROC curves of the six machine learning algorithm models in predicting the risk of LNM in gastric NEN patients. The RF (random forest) model showed the best performance with an AUC of 0.81.

Six models using machine learning algorithms were employed to build a predictive model. The training and internal validation sets were employed to train the models, and the average precision of the machine learning models was greater than 0.74, indicating good predictive ability. The RF model had the highest accuracy in predicting the risk of gastric NEN LNM occurrence and also showed the best performance with an AUC (Fig. 3) of 0.81, a sensitivity of 0.76, a specificity of 0.82, and an accuracy of 0.78 (Table 3).

**Table 3:** Summary of the performance of the machine learning models. KNN, k-nearest neighbor algorithm; SVM, support vector machine; LR, logistic regression model.

Model	Accuracy	Sensitivity	Specificity	AUC
Random Forest	0.782 (0.682, 0.845)	0.765 (0.665, 0.828)	0.824 (0.672, 0.866)	**0.810(0.782, 0.870)**
Decision Tree	0.773 (0.661, 0.869)	0.739 (0.692, 0.803)	0.889 (0.781, 0.924)	0.730 (0.693, 0.754)
Naive Bayes	0.773 (0.652, 0.845)	0.734 (0.696, 0.793)	**0.920(0.811, 0.982)**	0.790 (0.767, 0.845)
KNN	0.605 (0.501, 0.676)	**0.853(0.768, 0.901)**	0.506 (0.498, 0.532)	0.800 (0.681, 0.826)
SVM	0.782 (0.681, 0.884)	0.753 (0.682, 0.883)	0.867 (0.746, 0.901)	0.770 (0.659, 0.881)
LR	**0.798(0.701, 0.854)**	0.776 (0.601, 0.828)	0.853 (0.771, 0.893)	0.770 (0.661, 0.820)

Compared to traditional statistical analysis, which admits theoretically relevant parameters into the pre-designed model, machine learning can admit numerous variables and discern patterns from large datasets [19,20]. In this study, all extracted demographic and clinical characteristics from the SEER database were used as the features, excluding race and marital status, to optimize the predictive power of the machine learning models. Logistic regression models do not need to assume the data distribution in advance, avoiding the problems caused by the inaccurate assumption of distribution, are very robust to small noise in the data, and are not affected by slight multicollinearity. Due to the unbalanced samples, the RF model based on the AUC was selected as the best prediction model in this study.

### 3.4 Model Interpretation

**Fig. 4:**
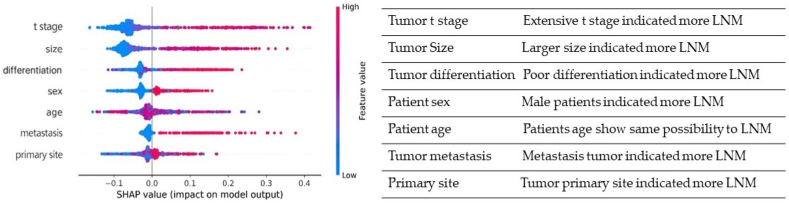
Summary plots for SHAP values in the RF model. The x-axis represents the impact of the feature on the model output for the patient with LNM. Features along the y-axis are arranged in the order of importance, which is given by the mean of their absolute Shapley values.

Fig. 4 depicts the SHAP summary plot, which consists of seven features sorted by their impact on metastatic occurrence. We examined the effects of the T stage, tumor size, degree of differentiation, and sex, which were the top four variables in the RF model affecting the predicted output.

## 4 Discussion

With the increasing incidence of gastric NENs, more attention is being paid to the prognosis of these tumors. LNM plays a crucial role in the prognosis and survival of gastric NEN patients [21] and is a determining factor concerning the choice of endoscopic or surgical treatment for intermediate tumors (11−20 mm) [22]. EMR or ESD are recommended for type I and type II gastric NENs between 1 and 2 cm, excluding cases of muscular invasion and suspicious perigastric lymph nodes at EUS and surgical resection (partial gastrectomy with lymph-node picking or resection) and when suspicious perigastric lymph nodes can be observed [[23], [24], [25]]. However, current guidelines did not provide detailed information about the risk evaluation of LNM and when a lymphectomy should be performed [[26], [27], [28], [29], [30]]. Additionally, research about the LNM of gastric NENs is still limited, thus we aimed to identify risk factors of LNM and use six machine learning algorithms to creative accurate predictive models. The predictive model's SHAP summary plot revealed that size, T stage, degree of differentiation, age, and M stage are important predictors of LNM in the algorithm.

The Eighth American Joint Committee on Cancer (AJCC) included tumor size in the T stage of GNETs and defined the T1 stage as invading the lamina propria or submucosa with a tumor less than 10 mm, while defining the T2 stage as invading the muscularis propria or a tumor size more than 10 mm [31]. Including the tumor size in the stage enhances the AJCC TNM's prognostic performance [32]. Additionally, multiple studies confirmed that tumor size is a powerful predictor of tumor prognosis and is strongly related to the status of lymph nodes [22,[33], [34], [35], [36]]. Zhou et al. pointed out that patients had a higher risk of LNM at 3.96 and 17.38 with a tumor size of 11–20 mm and more than 20 mm, respectively, compared to a tumor less than 10 mm in T1 and T2 gastric NENs [22]. This phenomenon can be explained by larger tumors typically associated with deeper invasion and a higher macroscopic grade [35]. Additionally, tumor size is correlated to the Ki-67 index, an important indicator distinguishing the tumor grade [37].

Numerous studies confirmed that the T stage is an independent predictor of LNM in various cancers [38,39]. The T stage represents the depth of tumor invasion and as expected, the risk of LNM increased as the T stage increased. Tumors with a higher T stage seem to more easily invade the surrounding tissues. Endoscopic therapy may be an alternative management strategy to surgical therapy for tumors confined to the mucosa and the submucosa. However, surgical management is the preferred option when the tumor penetrates the muscularis or deeper (T2 or higher), regardless of the tumor size [23,40,41]. Tumors with distant metastasis are defined as the M1 stage, meaning that the tumor has achieved an advanced stage (stage IV) and typically have more aggressive biological behavior. It has been reported that the incidence of LNM for patients with stage IV was 100 %, whereas it was <10 % for patients with stage I [42]. The incidence and extent of LNM positively correlate with T and M stages [43]. The degree of differentiation is correlated with tumor prognosis and LNM [44,45] and as a pathological risk factor, in part accounts for tumor biology. A large retrospective study reported that the degree of differentiation correlated with each component of the TNM system and the risk of LNM for each T stage [46]. The results also revealed that male patients had a higher risk of LNM, which is consistent with Chung's research which showed that patients were more commonly male, older, with deeper invasion, and a higher risk of LNM [47]. Our research also suggests that age is an important predictor. The prognostic value of age in gastric tumors is still controversial, but most studies reported a higher risk of metastasis in younger patients [[48], [49], [50]]. As age increases, lymph node stromal cells gradually degenerate, and lymph node morphology becomes unstable [51]. Additionally, older patients have a smaller lymph node cortex and medulla in the progressive degenerative process, eventually leading to inactive forms of LNM [52]. The effect of age on LNM may be explained by this mechanism.

The present study combined the large dataset of the SEER registry with the powerful tool of machine learning to develop an effective predicting tool of LNM for gastric NENs. The SEER database, as an authoritative source for cancer statistics in the United States, includes data on roughly 28 % of the country's population, which allows for the comprehensive assessment of gastric NENs' clinicopathologic characteristics to build machine learning-based predictive models [53]. Additionally, the SEER program requires that cancer registries comply with strict quality control standards regarding the identification of cases and the quality of the data collected [54]. The predictive ability of the machine learning algorithms was further evaluated using data from 119 patients from another hospital, confirming that our model has a good predictive ability.

Compared with traditional statistical analysis, machine learning uses a different means of data processing. In traditional statistical approaches, only theoretically relevant parameters or significant univariate parameters are admitted into the pre-designed model, whereas machine learning can admit numerous variables and discern patterns from large datasets. Then, patterns are codified into a mathematical model that is applied to newly obtained data for further validation [19]. The application of machine learning may reveal hidden knowledge that conventional statistical analysis fails to detect [55]. In addition, machine learning algorithms can analyze various data types (such as demographic data, imaging data, and laboratory findings) and are more flexible and scalable compared to traditional predicting models [56]. By combining the large dataset of the SEER registry with the powerful tool of machine learning, we evaluated six machine learning algorithms, showing that random forest had the best performance (AUC = 0.81) and high accuracy (0.782) when using our hospital data as external validation. In addition, we excluded patients with incomplete information to avoid incorrect imputation.

This study has some limitations. First, some important histopathological information is not available in the SEER dataset, such as the mitotic counts, Ki-67 index, vascular invasion, and neural invasion, which are important for tumor grading and tumor prognosis [57,58]. Second, patients with gastric NENs were not classified according to clinical subgroups (three types according to etiology, in which type 3 lesions have the poorest differentiation and are accompanied by poor outcomes). Third, this study was retrospective, and only those patients with complete data were selected and analyzed, thus there is a risk of selection bias [59,60]. In addition, our external validation set comprises a single center. Prospective research with a large number of patients is expected to explore the risk factors of LNM.

## 5 Conclusion

Six machine learning algorithms were utilized to the demographic characteristics of patients with gastric NENs to establish predictive models of LNM, revealing that T stage, tumor size, degree of differentiation, and sex are independent risk factors for predicting LNM. Random forest exhibited the best predictive ability, thus has potential clinical application value.

## Funding

The science and technology plan of Suzhou city (SKY2021038).

## Institutional review Board statement

This study was approved by the Ethics Committee of the First Affiliated Hospital of Soochow University (Number: 2022-232).

## Informed consent statement

Informed consent was obtained from the subjects involved in the study from the First Affiliated Hospital of Soochow University.

## Data availability statement

Data will be made available on GitHub and the website is: https://github.com/shulu512162/test/blob/main/ML.R.

## CRediT authorship contribution statement

**Lu Liu:** Writing – original draft. **Wen Liu:** Writing – original draft. **Zhenyu Jia:** Writing – original draft. **Yao Li:** Data curation. **Hongyu Wu:** Software. **Shuting Qu:** Software. **Jinzhou Zhu:** Software. **Xiaolin Liu:** Writing – review & editing. **Chunfang Xu:** Writing – review & editing.

## Declaration of competing interest

The authors declare that they have no known competing financial interests or personal relationships that could have appeared to influence the work reported in this paper.
